# Neurological prognostication after cardiac arrest

**DOI:** 10.1186/1757-7241-16-10

**Published:** 2008-09-15

**Authors:** Hans Friberg

**Affiliations:** 1 Department of Anesthesiology and Intensive Care, Lund University Hospital, Sweden

## 

The care of the comatose cardiac arrest patient and his/her relatives in the ICU constitutes a major challenge for intensivists. Out-of-hospital cardiac arrest (OHCA) affects more than 275.000 Europeans yearly; survival rates are disturbingly low and have until recently remained unchanged [1]. During the last several years, significant efforts have been made to improve the quality of prehospital care, with a focus on more effective chest compressions. Postresuscitation care in the hospital was long ago identified as the "missing link" in the chain of survival, but a change in provision of care has occurred, as illustrated by the national Norwegian survey, published in the Scandinavian Journal of Trauma, Resuscitation and Emergency Medicine [2]. Three years have passed since the telephone survey, but 80% of the hospitals in Norway had already introduced routine therapeutic hypothermia (TH) at that time. Today, almost every emergency hospital in all of Scandinavia routinely treats comatose cardiac arrest patients of cardiac origin with TH, and many have implemented a standardised treatment protocol as suggested [3].

This novel, active treatment of cardiac arrest patients in the ICU, as opposed to the former supportive approach, has given the patients' relatives increased hope, presenting new challenges to intensivists. The questions of when and how to inform relatives of the prognosis of an individual patient and of what prognostic tools should be used are still under debate. An evidence-based review on the prediction of outcome in comatose surviviors after cardiopulmonary resuscitation was recently published, but it remains unclear whether these results can be applied to patients treated with hypothermia [4]. The survey by Busch et al. [2] unsurprisingly shows that there is a great deal of insecurity and even a lack of knowledge in the medical community regarding prognostication after cardiac arrest. The introduction of TH has changed the prerequisites for prognostication. The cooled patient is sedated and intermittently paralysed during treatment, and at the same time, the patient's metabolism is lowered by approximately 30%, affecting drug turnover. Moreover, a lowered temperature over an extended period of time may affect brain functions in an unpredictable way. Many intensivists can testify to very late recoveries in individual comatose, hypothermia-treated patients. However, until we know more, great caution should be employed when interpreting prognostic measures in these patients, and, even more importantly, the timing of prognostication should be postponed. However, this necessary change has not yet been reflected in clinical practise; a majority of hospitals in the survey by Busch et al. perform prognostication within 48 hours after hospital admission. It is encouraging, though, that a multidisciplinary approach was applied in prognostication in a large majority of hospitals in the survey, involving intensivists, internists and neurologists.

This analysis leads to the following statements. First, neurological prognostication after cardiac arrest should routinely be performed as a joint task, preferably together with a neurologist, and it should be carried out using a standardised protocol. Second, prognostication must be postponed in comatose cardiac arrest patients and should be performed no earlier than 48–72 h after resumption of normothermia. In addition, sedation should be withdrawn prior to a decisive neurological examination. If in doubt, a new evaluation should be performed daily, and, if no improvement is seen after three days, termination of active treatment should be considered. Third, a clinical neurological examination is still the basis for prognostication [4,5], on which 100% of Norwegian hospitals in the present survey agree. Moreover, at least in our centre, a decision to withdraw treatment may only be taken when a neurological examination is corroborated by at least one objective measure. Such measures include a pathological electroencephalographic (EEG) pattern at normothermia [6], a somatosensory evoked potential (SSEP) investigation with a bilateral lack of cortical response [7,8], and high and increasing levels of the brain damage marker neuronspecific enolase (NSE) at 24–48 h post arrest [[8,9], own results, to be published]. Hence, a score on the reaction level scale (RLS) of 7 or 8, corresponding to a value of 3 or 4 on the Glasgow Coma Scale (GCS), in combination with one or more objective parameters of severe brain damage provides the basis for a decision to withdraw treatment in our ICU. The difficulties and pitfalls of prognostication after cardiac arrest, induced hypothermia and more will be discussed at the upcoming 3^rd ^International Hypothermia Symposium in Lund, Sweden, 2–5 Sept. 2009. Please find more information on our website .

Finally, we agree with Busch et al. that international evidence-based guidelines for the prognostication and follow-up of hypothermia-treated cardiac arrest patients are required. In fact, this work is now being incorporated into the guidelines being established by the 2010 International Liaison Committee on Resuscitation (ILCOR).

## Competing interests

The author declares that they have no competing interests.
